# A Challenging Issue in the Etiology of Speech Problems: The Effect of Maternal Exposure to Electromagnetic Fields on Speech Problems in the Offspring

**Published:** 2015-09-01

**Authors:** S. Zarei, S. M. J. Mortazavi, A. R. Mehdizadeh, M. Jalalipour, S. Borzou, S. Taeb, M. Haghani, S. A. R. Mortazavi, M. B. Shojaei-fard, S. Nematollahi, N. Alighanbari, S. Jarideh

**Affiliations:** 1 Speech and Language Pathology Department, School of Rehabilitation, Shiraz University of Medical Sciences, Shiraz, Iran; 2 President of the Ionizing and Non-ionizing Radiation Protection Research Center (INIRPRC); Professor of Medical Physics in the School of Medicine of Shiraz University of Medical Sciences, Shiraz, Iran; 3 Department of Medical Physics and Engineering, School of Medicine, Shiraz University of Medical Science, Shiraz, Iran; 4 Ph.D candidate at the Ionizing and Non-ionizing Radiation Protection Research Center (INIRPRC), Shiraz University of Medical Sciences, Shiraz, Iran; 5 Ionizing and Non-ionizing Radiation Protection Research Center (INIRPRC), Shiraz University of Medical Sciences, Shiraz, Iran; 6 Medical Student at Student Research Committee, School of Medicine, Shiraz University of Medical Sciences, Shiraz, Iran; 7 Master Student at the Biostatistics Department, School of Medicine, Shiraz University of Medical Sciences, Shiraz, Iran; 8 Occupational Health Department, School of Health, Shiraz University of Medical Sciences, Shiraz, Iran

**Keywords:** Speech Problem, Maternal Exposure, Pregnancy, Electromagnetic Fields, Mobile Phones

## Abstract

**Background:**

Nowadays, mothers are continuously exposed to different sources of electromagnetic fields before and even during pregnancy.  It has recently been shown that exposure to mobile phone radiation during pregnancy may lead to adverse effects on the brain development in offspring and cause hyperactivity. Researchers have shown that behavioral problems in laboratory animals which have a similar appearance to ADHD are caused by intrauterine exposure to mobile phones.

**Objective:**

The purpose of this study was to investigate whether the maternal exposure to different sources of electromagnetic fields affect on the rate and severity of speech problems in their offspring.

**Methods:**

In this study, mothers of 35 healthy 3-5 year old children (control group) and 77 children and diagnosed with speech problems who had been referred to a speech treatment center in Shiraz, Iran were interviewed. These mothers were asked whether they had exposure to different sources of electromagnetic fields such as mobile phones, mobile base stations, Wi-Fi, cordless phones, laptops and power lines.

**Results:**

We found a significant association between either the call time (P=0.002) or history of mobile phone use (months used) and speech problems in the offspring (P=0.003). However, other exposures had no effect on the occurrence of speech problems. To the best of our knowledge, this is the first study to investigate a possible association between maternal exposure to electromagnetic field and speech problems in the offspring. Although a major limitation in our study is the relatively small sample size, this study indicates that the maternal exposure to common sources of electromagnetic fields such as mobile phones can affect the occurrence of speech problems in the offspring.

## Introduction

Generation, transmission and applications of electricity have been increased rapidly as an essential part of the modern life. Over the past decades technology has significantly improved the communication, and nowadays, telecommunication is playing an important role in decreasing the distance among people around the world. Now, mothers are continuously exposed to different sources of electromagnetic fields such as mobile phones, mobile base stations, and Wi-Fi before and even during pregnancy.  Recent findings show that exposure to electromagnetic radiation in radiofrequency range (EMR-RF) emitted by mobile phones during pregnancy may lead to adverse effects on the brain development in offspring which causes hyperactivity. Researchers have shown that behavioral problems in laboratory animals which have a similar appearance to ADHD are caused by intrauterine exposure to mobile phone radiations[1]. Over the past years, our laboratory has focused on studying the health effects of exposure of laboratory animals and humans to some common and/or occupational sources of electromagnetic fields such as mobile phones[2-9] and their base stations[10], mobile phone jammers[11], laptop computers[12], radars[3], dentistry cavitrons[13] and MRI[14, 15].  The purpose of this study was to investigate whether the maternal exposure to different sources of electromagnetic fields affect on the rate and severity of speech problems in their offspring.

## Materials and Methods

This cross-sectional study was performed from June till December 2014 in Shiraz, Iran. Mothers of 35 healthy children (control group) and 77 children aged 3-5 year and diagnosed with speech problems who had been referred to a speech treatment center in Shiraz, Iran were interviewed. Due to very limited availability, mothers were selected using convenient sampling. Therefore, all mothers attending the clinics covered in this study on the day of interview and gave written consent, were interviewed. These mothers were asked whether they had exposure to different sources of electromagnetic fields such as mobile phones, mobile base stations, Wi-Fi, cordless phones, laptops and power lines. A semi-structured questionnaire designed to assess all possible exposures to electromagnetic fields was used for information recording. This study was approved by the Medical Ethics committee of Shiraz University of Medical Sciences. Data were analyzed by using SPSS (ver 19.0). A P value of less than 0.05 was considered as significant. 

## Results

Demographic data for study participants are summarized in the Table 1. The mean age of mothers at the time of delivery and interview were 25.78±5.15 and 29.77±5.72 years, respectively. About 94% of the participants were housewives. The proportion of consanguineous marriage in the participants was 43.8%. The mean number of pregnancies and normal children in each family were 1.84±0.85 and 1.54±0.71, respectively. All mothers were non-smokers but 2.7% had the history of addiction. For dental radiography, this rate was only 0.9%. Regarding exposure to ionizing radiation, only 3.6% had the history of non-dental radiography during pregnancy. In case of exposure to non-ionizing radiation, only 23% of the mothers had not used mobile phones during pregnancy. The mean daily call time was about 20 min and less than 8% had used their mobile phone for durations longer than 60 min. Using cordless phones during pregnancy was more frequent and 44% had used these telecommunication devices. In this study 51.4% of the participants had the history of using CRTs during pregnancy. Exposure to extremely low frequencies induced by high tension power lines was only reported by 5.3% of the mothers.

**Table 1:** Characteristics of the mothers participated in this study.

**Characters**	**Frequency ** **(Percentage)**
**Age at the Time of Interview**	
18-20 years	4 (3.6%)
21–25 years	27 (24.6%)
26–30 years	28 (29.0%)
31-35 years	36 (17.1%)
>35 years	15 (13.6%)
**Age at the Time of Delivery**	
18-20 years	15 (13.9%)
21–25 years	40 (37.0%)
26–30 years	38 (35.2%)
31-35 years	9 (8.3%)
>35 years	6 (5.6%)
**Consanguineous marriage**	
Consanguineous marriage	49 (44.5%)
Non-Consanguineous marriage	61 (56.5%)
**Job**	
Housewife	104 (94.5%)
Nurse	4 (3.6%)
Student	1 (0.9%)
Other jobs	1 (0.9%)

We found a significant association between either the call time (P=0.002) or history of mobile phone use (months used) and speech problems in the offspring (P=0.003). However, average duration of daily call time during pregnancy had no effect on the occurrence of speech problems. This study could not show a significant association between cordless phone use and speech problems in the offspring (P=0.528). Furthermore, there was no association between CRT use and speech problems in the offspring (P=0.990). 

## Discussion

To the best of our knowledge, this is the first study to investigate a possible association between maternal exposure to electromagnetic field and speech problems in the offspring. Altogether, findings of this study showed a significant association between exposure parameters of mobile phones such as the call time or history of mobile phone use and the occurrence of speech problems in the offspring. However, we could not show any association between exposure to cordless phones or CRTs and the occurrence of speech problems. This difference can be due to the different exposure patterns. Mobile phones are usually held near the body when being used. More specifically, the user’s brain is in close proximity to mobile phone during talks. It is worth mentioning that  some studies has provided evidence that low doses of ionizing radiation to the pregnant mother’s head is associated with disorders in the offspring such as low-birth-weight. Interestingly, it is hypothesized that the hypothalamus-pituitary-thyroid axis is to some extent involved in this causal pathway[16]. On the other hand, the peak power of mobile phones is up to 2 watts. This difference can explain the reason for lack of any association between exposure to cordless phones and speech problems in this study.

At a general view, findings obtained in our study are in line with the results reported by Taylor and his team at Yale University who showed that behavioral problems in mice which are similar to ADHD can be caused by maternal exposure to mobile phones during pregnancy[1, 17]. They exposed pregnant mice to radiofrequency radiation emitted by a muted mobile phone (talk mode but muted) that was placed above their cage during the examination period. Taylor and his colleagues reported that the offspring that were exposed to mobile phone radiation in their intrauterine life, showed more hyperactivity and reduced memory capacity compared to unexposed control group. Interestingly, Taylor hypothesized that the current rise in behavioral disorders in human children may be to some extent due to fetal exposure to mobile phone radiations[1].

Although a major limitation in our study is the relatively small sample size, this study indicates that the maternal exposure to common sources of electromagnetic fields such as mobile phones can affect the occurrence of speech problems in the offspring. 
